# Assessment of drug-related problems among type 2 diabetic patients on follow up at Hiwot Fana Specialized University Hospital, Harar, Eastern Ethiopia

**DOI:** 10.1186/s13104-019-4760-8

**Published:** 2019-11-27

**Authors:** Haymen Abdulmalik, Yohannes Tadiwos, Nanati Legese

**Affiliations:** School of Pharmacy, College of Health and Medical Sciences, Haramaya University, P.O. Box 235, Harar, Ethiopia

**Keywords:** Diabetes mellitus, Drug therapy problem, Hiwot Fana Specialized University Hospital

## Abstract

**Objectives:**

To assess the drug-related problem among patients with type 2 diabetes at Hiwot Fana Specialized University Hospital.

**Results:**

In this study, a total of 148 patient medication records were included. More than half, 83 (57.4%) were men and the rest 65 (42.6%) were women. The mean age of the study participants was 51.26 ± 7.08. Around one-third (74.3%) of the participants had urban residency. A total of 127 drug-related problems were identified, of which dosage too low was the most common type of DRP encountered, 46 (36.2%), followed by unnecessary drug therapy, 25 (19.7%) and ineffective drug therapy, 25 (19.7%). 95 (64.2%) of the patients had at least one drug-related problem. Among patients with DRP, more than half of them, 59 (62.1%) had a single DRP. Out of the total participants, 85 (57.4%) of them were taking one anti-diabetic medication and 63 (42.6%) of them dual anti-diabetic medications. Only half of the patients have attained the desired FBG level. There was no patient who had experienced more than two types of drug-related problems at a time. Less than 10% of patients were taking five or more drugs at a time.

## Introduction

Type 2 diabetes mellitus (DM) is associated with an increase in blood glucose resulting from a defect in insulin secretion and insulin action and with a resulting disturbance in carbohydrate, fat and protein metabolism [1–4]. Both the number of cases and the prevalence of diabetes have been steadily increasing over the past few decades [5, 6].

For the treatment of T2DM, varieties of drugs with a different mechanism of action are used to maintain glycemic control [7]. Patients with T2DM are at risk of drug-related problems (DRP), which can happen at any step during the treatment process [8–10] and it affects the therapeutic outcome [3, 11]. Problems associated with drug use are many and are classified into different system by different researchers [12] and include untreated condition, inappropriate choice of drugs, unnecessary drug therapy, failure to receive drug, discrepancies between prescribed and actual regimens, overdose, adverse drug reactions (ADRs), inappropriate medication prescribing, and drug use, drug interactions [8].

There exists a problem in the prescribing, dispensing and usage of drugs [8] resulting in the unwanted effect of medications [13]. This with the ineffectiveness of the drugs chosen makes the management of DM challenging [12]. Multiple co-morbidities, increasing age and the number of medications taken by DM patients increase the risk of DRP [6, 10, 14, 15]. Other factors such as renal impairment, poor lipid control, cardiovascular disease, and the duration of hospital stay also increase DRP risk [16, 17]. DRP is a worldwide health problem that compromises the quality of life, increase hospitalization, increase overall health care cost and mortality [8, 9, 12, 18, 19].

This study tries to assess DRPs among T2DM patients on follow up at Hiwot Fana Specialized Hospital (HFSUH). Identifying the DRP and optimizing drug treatment helps to facilitate the rational use of the drugs and help patients to achieve their goals of therapy.

## Main text

### Methodology

#### Study design and study setting

A facility-based retrospective cross-sectional study was conducted at HFSUH by reviewing the medical records of T2DM outpatients on chronic follow up from July–December 2018 G.C at HFSUH.

#### Study population

Patients diagnosed with T2DM aged 18 years or older of both gender and receiving at least one anti-diabetic medication and those who had been on treatment for at least 3 months at HFSUH during the data collection period were included in the study. Patients with missing or incomplete data were excluded.

#### Sample size determination and sampling technique

The sample size was 148 calculated using a single population proportion formula, with a prevalence of 45.9% based on the previous study done in Addis Ababa, at Tikur Anbessa Specialized Hospital (TASH) [20] and the total number of T2DM on follow up at HFSUH was 211. The patient medical record (PMR) was selected using a simple random sampling technique.

#### Data collection method and procedure

The record of patients with T2DM patients on chronic follow-up from July–December 2018 G.C at HFSUH was identified until the targeted sample size was achieved. The required information was collected by the principal investigator from the PMR using a structured data collection checklist. Cipolle’s method of DRP classification system was used together with the Ethiopian treatment Guideline and standards of medical care for diabetes.

#### Data processing and analysis

Data were checked for completeness by the principal investigator (PI) on a daily basis during collection before actual analysis. The analysis was done using statistical software for social sciences (SPSS) 20.

#### Operational definition

DRP: Refers to any unwanted incident related to medication therapy that actually or potentially affects the desired goals of treatment [12].

Cipolle’s method: It classifies DRPs into seven major groups as follows: These include: including unnecessary drug therapy, needs additional drug therapy, ineffective drug therapy, dosage too low, adverse drug reaction, dosage too high and noncompliance [21].

#### The DRPs were defined as follows:

Dosage too high: If the prescribed dose was too high than the recommended dose by standard treatment guideline of Ethiopia [22].

Dosage too low: If the prescribed dose was less than the recommended dose by standard treatment guideline of Ethiopia [22].

Unnecessary drug therapy: If there is duplication of therapy or unwanted addition of medication or if the patient doesn’t have an indication for adding another medication.

Needs additional drug therapy: If a patient was inadequately medicated with his/her blood glucose not within the target range (80–130 mg/dL**)**, this was classified as needs additional drug therapy.

ADR: Adequate doses resulting in unpleasant or harmful reactions.

Ineffective drug therapy: The inappropriate use of drugs according to standard treatment guideline of Ethiopia [22].

### Result

In this study, a total of 148 T2DM PMR were included. More than half, 83 (57.4%) were males and the rest 65 (42.6%) were females. The mean age of the study participants was 51.26 ± 7.08. Around one-third (74.3%) of the participants had urban residency. More than half of the patients (58.1%) had a duration of illness 1 up to 5 years. The overall mean (± SD) average value of FBG for the last three consecutive visits was 129.14 ± 31.621. Half of the participants (50%) meet the intended glycaemic FBG target and 66 (44.6%) of them recorded above > 130 mg/dL. Out of the total study participants, 51 (34.5%) had comorbid medical problems. The most common comorbid disease was hypertension 30 (20.3%), followed by CHF 7 (4.7%). Only 9 (6.1%) participants had developed chronic diabetes complications and of these, 7 (77.8%) of them encountered diabetic peripheral neuropathy (Table 1).

**Table 1:** Socio-demographic and clinical characteristics of ambulatory patients with type 2 diabetes on follow up from July to December 2018 at HFSUH, Harar, Ethiopia

Variables	Frequency	Percentage
Age		
20–40	11	7.4
41–60	124	83.8
≥ 61	13	8.8
Total	148	100
Sex		
Male	83	57.4
Female	65	42.6
Total	148	100
Residency		
Urban (Harar)	110	74.3
Rural	38	25.7
Total	148	100
Duration of diabetes		
< 1 year	33	22.3
1–5 years	86	58.1
6–10 years	22	14.9
> 10 years	7	4.7
Total	148	100
Average FBG (mg/dL)		
< 80	8	5.4
80–130	74	50
> 130	66	44.6
Total	148	100
Comorbidity		
Hypertension	30	20.3
CHF	7	4.7
Dyslipidemia	4	2.7
IHD	3	2.03
Others^a^	7	4.7
Total	51	34.5
Complication		
Nephropathy	7	4.7
Retinopathy	2	1.4
Total	9	6.1

^a^COPD and asthma

Out of the total participants, 85 (57.4%) of them were taking one anti-diabetic medication, from these metformin and basal insulin accounts for 55 (37.2%) and 24 (16.2%) respectively. Dual anti-diabetic medications were used in 63 (42.6%) cases and metformin with Glibenclamide were used for 41 (27.7%) cases, followed by metformin with basal insulin in 19 (12.8%) cases. A total of 113 drugs were found in the patient chart for chronic comorbid conditions, Enalapril 25 (22.1%), Nifedipine 16 (14.2%), hydrochlorothiazide 15 (13.3%) and aspirin 14 (12.4%) were most frequently prescribed medications. Only 12 (8.1%) of the participants were taking 5 drugs and above (Table 2).

**Table 2:** Prescribed medications for ambulatory patients with type 2 diabetes on follow up at HFSUH from July–December 2018, Harar, Ethiopia

Medications	Frequency	Percentage
Anti-diabetic medications		
Metformin	55	37.2
Metformin + glibenclamide	41	27.7
Basal insulin	24	16.2
Metformin + basal insulin	19	12.8
Glibenclamide	6	4.1
Glibenclamide + basal insulin	3	2
Total	148	100
Other medications		
Enalapril	25	22.1
Nifedipine	16	14.2
Hydrochlorothiazide	15	13.3
Aspirin	14	12.4
Atorvastatin	10	8.8
Metoprolol	8	7.1
Anti-asthmatics	7	6.2
Furosemide	7	6.2
Others^a^	11	9.7
Total	113	100
Total number of drug per patient		
< 5	136	91.9
≥ 5	12	8.1
Total	148	100

^a^Spironolactone, amitriptyline and benzyl penicillin

From the total 148 PMRs, 95 (64.2%) patients had at least one DRP identified and a total of 127 DRPs were identified, which is 0.9 DRPs per patient. Among patients with DRP, more than half of them, 59 (62.1%) had a single DRP and 36 (37.9) of them had double DRP. Dosage too low was the most common type of DRP encountered, 46 (36.2%), followed by unnecessary drug therapy, 25 (19.7%) and ineffective drug therapy, 25 (19.7%) (Table 3).

**Table 3:** Drug related problems among ambulatory patients with type 2 diabetes on follow up from July to December 2018 at HFSUH, Harar, Ethiopia

DRPs	Frequency	Percentage
Number of patients with DRP		
Single DRP	59	62.1
Double DRPs	36	37.9
Total	95	100
Types of DRPs		
Dosage too low	46	36.2
Metformin + glibenclamide	10	7.9
Meftormin + basal insulin	13	10.2
Metformin	18	14.2
Basal insulin	5	3.9
Ineffective drug	25	19.7
Basal insulin alone	8	6.3
Metformin alone	1	0.8
Glibenclamide	3	2.4
Metformin + basal insulin	5	3.9
Metformin + glibenclamide	6	4.7
Basal insulin + glibenclamide	2	1.6
Unnecessary drug therapy	25	19.7
Basal insulin as unnecessary	10	7.9
Glibenclamide as unnecessary	15	11.8
Need for additional drug therapy	17	13.4
Taking metformin alone	12	9.4
Taking metformin and glibenclamide	3	2.4
Taking glibenclamide	2	1.6
Adverse drug reaction	12	9.4
Metformin	2	1.6
Glibenclamide	6	4.7
Basal insulin	4	3.1
Dosage too high	2	1.6
Basal insulin	2	1.6
Total DRPs	127	100

### Discussion

In the current study, a total of 127 DRPs were identified from 148 PMR, which is 0.9 DRPs per patient. 64.2% of the patients had a DRP problem identified. This is lower than the result obtained in Wolaita Soddo, southern Ethiopia, which showed that 83.1% of T2DM patients had at least one DRP with the mean number of 1.8 ± 0.751DRPs [2] and with Danish study which shows an average of 4.1 DRPs per patients [23]. The discrepancy with these studies could be due to the use of different data collection methods since the study conducted in Wolaita Soddo also include interviewer-administered questionnaire and difference in the study protocol since the Danish study uses Problem Intervention Documentation (PI-DOC). Socio-demographics and co-morbid conditions of study patients could also affect the DRP. The individuals with co-morbid conditions in the current study account for 34.5% of the study population, which is lesser than the study done in Wolaita Soddo (56%). Another result from Malaysia’s study also showed a higher average DRP (2.37 ± 1.40) than the current study [17]. This difference could be attributed to the difference in the study population, since the study in Malaysia only involves T2DM patients with dyslipidemia and also due to the difference in the study protocol, with the Malaysian study using Pharmaceutical Care Network Europe (PCNE). Also, the lower level of DRP reported in the current study could also be attributed to the lesser number of medications taken by the patients, with only 8.1% of the patients taking more than five medications at a time.

The common DRP identified in the current study were dosage too low (36.2%), ineffective drug (25%) and unnecessary drug therapy (25%). This is different from a study done in Malaysia, where the two most common DRP classifications identified in the current study were “potential interaction (18%)” and “drug not taken or administered at all (14.3%)” [17]. Inappropriate use of medicine (26.9%) and inappropriate choice of medicine (9.1%) were the commonly documented DRP in a Danish study [23]. The difference in the frequency of various DRPs from the current study could be attributed to the difference in the methodology (such as a medical review or interview technique) and types of DRP classification (such as Cipolle, PCNE or PI-Doc system) used. The DRP problems could also occur due to the problem of inadequate follow up at HFSUH and could be due to the absence of some laboratory findings such as hemoglobin A1C (HbA1C). Such inadequate follow up could be due to the higher workload on the working health care practitioners at HFSUH. One way of reducing DRP is involving a clinical pharmacist, who may assess DRPs in different settings: in hospital multidisciplinary teams, in nursing homes and in primary care [12]. The involvement of clinical pharmacists as a member of the healthcare team helps in the identification and prevention of DRPs which will help to rationalize drug therapy, achieve better therapeutic outcomes and improved the quality of patient care [3].

Dosage too low type of DRPs constituted 36.2% of the total DRPs in the current study. This report was higher than the study done in Indonesia which was 7.3% [6], a study that was done in Wolaita Soddo (26.75%) [2] and the study done in southwest Ethiopia (Jimma specialized hospital) which covered 15.8% [24]. Such a high prevalence of dosage too low in the current study has been associated with a higher number of T2DM patients (44.6%) not attaining the desired fasting blood glucose (FBG). This difference could due to the difference in the study population between the current study and the study done in Indonesia and Southwest Ethiopia.

From the total 148 PMRs, 95 (64.2%) of the patients had one or two DRPs identified. Among those, more than half of the patients, 62.1% of them had a single DRP. This result is lower than the result obtained in Malaysia, which shows that 90.5% of the patients had at least one DRP [10], the study done in India, which shows that 71% of the patients had at least one DRP [3] and the study that was done in southwest Ethiopia, which revealed that 82% of the participants had at least one drug-related problem [24]. This variation across the studies can be attributed to the fact that the study population in Malaysia, India and southwest Ethiopia is T2DM patients with hypertension problem, which is different from the current study. The difference in DRP identified could also be due to the different study methods used by these studies. There exists also a difference in the number of medications used by the patients. In the current study, only 8.1% of the study population uses more than 5 medications which is less than the result obtained in southwest Ethiopia, which shows that 34% of them use more than 5 medications, which will increase the risk of DRPs.

In the current study, ADR (9.4%) and dosage too high (1.6%) were less frequently occurring DRPs. This is similar to a study in southwest Ethiopia, where these two accounted for the less frequently occurring DRPs [24]. This is different from a study done in Nigeria, which showed ADR was the leading DRP 108 (35.3%) [11]. Such difference could be attributed to the difference in the age group of the study population, with the mean age of the current study is 51.26 ± 7.08 and that of the Nigerian study is 61.4 ± 12.8 [11]. The higher incidence of ADR could be attributed to the fact that likely hood of having DTPs increases as the age of respondents increases [2, 3]. The absence of laboratory findings such as liver function test and kidney function indicators in the PMR used to assess ADR could also be another reason why ADR was one of the less frequently experienced DRP in the current study. The less number of medications taken by the patients (8.1%) could also reduce the chance of drug interaction, reducing the chance of ADR.

### Conclusion

More than half of the participants had at least one DRP identified, with dosage too low, ineffective drug and unnecessary drug therapy being the common DRPs. Out of the 148 patients, only half of the patients have attained the desired FBG level. The present result underscored the need to promote pharmaceutical care at all levels of health care especially in chronic disease management to eliminate DRP and improve treatment outcomes. The involvement of clinical pharmacists in chronic follow up units is very important to reduce DRPs and they should work in collaboration with other health care professionals. Some laboratory findings used to assess ADR were missing, so better documentation is necessary for the betterment of treatment.

## Limitation of the study

The major limitations of the study were that the evaluation relied merely on the records of patients, for which all necessary information was not recorded such as laboratory value hence the DRP was difficult to determine. Since this study was a retrospective study, it doesn’t determine the DRPs that are associated with inappropriate use of the medications by the patients (doesn’t cover the DRP associated with adherence). This study is also a cross-sectional type and thus it did not investigate cause and effect relationship and also the small sample size makes it difficult to generalize the findings to the general population. Hence, further studies, which take these variables into consideration, will be needed to solve these limitations.

## Data Availability

The datasets used and/or analyzed during the current study are available from the corresponding author on reasonable request.

## Abbreviations

ADR: adverse drug reaction
DM: diabetes mellitus
DRPs: drug related problems
HFSUH: Hiwot Fana Specialized University Hospital
PMR: patients’ medical records
T2DM: type 2 diabetes mellitus
